# The rise of infodemiology and infoveillance during COVID-19 crisis

**DOI:** 10.1016/j.onehlt.2021.100288

**Published:** 2021-07-03

**Authors:** Steffen Springer, Michael Zieger, Artur Strzelecki

**Affiliations:** a SRH Wald-Klinikum Gera, Straße des Friedens 122, D-07548 Gera, Germany; b Department of Informatics, University of Economics in Katowice, Katowice 40-287, 1 Maja 50, Poland

**Keywords:** Google trends, COVID-19, Search engine data

## Abstract

We noticed an increase in the relative number of published papers on topics such as infoveillance, infodemiology and Google Trends. Collected PubMed data are from the period of January 2020 to March 2021 and were searched with the use of five keywords: infoveillance, infodemiology, Google Trends, diabetes and *in silico*. We compared an increase in the number of papers from PubMed with search interest expressed in Google Trends. Collected Google Trends data is from the same period, covering fifteen months starting January 2020 and were searched with the use of three search topics: coronavirus, lockdown and social distancing. The geographic setting for search engine users was worldwide. We propose a hypothesis that after increased interest in searches during the pandemic's initial months came an increased number of published papers on topics such as infoveillance, infodemiology and Google Trends.

## Highlights

• Google Trends data underline the importance and duration of the COVID-19 effects.
• PubMed data reveal an increase in the number of papers in infoveillance, infodemiology and Google Trends
• Current restrictions such as lockdown and social distancing are reflected in the number of internet searches

## 1 Introduction

Advances in computer technology since the 1980s have enabled the modelling of biochemical, molecular interactions, and physiological studies using computer programs [22,23]. The pseudo-Latin expression “*in silico*” dates back to 1987, when it was first introduced to describe artificial life [12]. Since then, the term has been used to model processes or developments with the help of computers and has evolved up to today's neuronal networks and artificial intelligence systems.

Today, the digital approach is widely used, for example, in digital epidemiology, which is based on digital data analysis [21]. Eysenbach introduced the term “infodemiology” for the internet-based approach and also defined “infoveillance” as a term [17]. The term infodemiology comes from information epidemiology, and infoveillance comes from information surveillance [8]. Digital epidemiology is epidemiology that uses digital data [28].

In recent years, extensive research has been conducted using data collected from Google Trends (GT), Google Flu Trends, and Google Cloud Healthcare API. More and more research uses GT [1]. Before the release of GT, early research was done on Google Flu Trends, which is a source of queries related to diseases [10]. Google Trends (GT) has been widely used as a study tool for a variety of medical indications [14]. GT is the source of reverse engineering data. It displays the content searched in Google, normalises the data according to the search frequency, and displays it in relative search volume. The data are divided into years and months and geographic regions. Researchers can compare up to five keywords using segmentation at a time. Research on GT can be divided into four areas - infectious diseases, mental health, other diseases and general population behaviour [19] -and mainly conduct seasonality [18].

The research gap we noticed includes the new circumstances that were caused by the COVID-19 pandemic. Studies with the use of GT have been already conducted for the last ten years. GT was used to study several infectious diseases, mental health, other diseases, and general population interest in health. None of the previous studies was on infectious disease, which became a global pandemic like COVID-19. We hypothesis that after announcing COVID-19 as a worldwide pandemic, the number of studies with the use of GT, *e.g.* about COVID-19 information interest, will increase.

Since the identified gap is a lack of studies, which would analyse the increase in infoveillance and infodemiology research, the objective of this work is to fulfil the identified gap. The general objective is to identify the possible rise in the number of papers on infodemiology and infoveillance during the COVID-19 crisis. The specific objectives are to assess the knowledge towards the increase in the *in silico* studies, identify the relative values in the increase, and compare the increase with interest in the general population behaviour during a pandemic time like lockdown and social distancing.

## 2 Methodology

In our study, we used a two-step methodology. First, we have collected data from the PubMed database on published papers containing specified keywords in the title/abstract. We have searched in PubMed for the following keywords: “*diabetes*”, “*Google Trends*”, “*in silico*”, “*infodemiology*”, “*infoveillance*”. We have selected “*infoveillance”* and “*infodemiology”* as important terms introduced by Eysenbach. “*In silico”* was chosen as well-established in literature and artificial intelligence systems term introduced by Langton in 1987 [12]. “*Google Trends*” is used in the title of the study to show that the research uses reversed engineered data. “*Diabetes*” was chosen as a comparative keyword, not related to the aforementioned keywords. It works as comparison data to show the relative increase for other keywords. It was used as a possible reference simply because it is a well-known term and a widespread disease. The data were away from the background noise with a sufficient search volume.

PubMed data were collected for the fifteen months indicated with the search terms of interest in the title and abstract. The search period was from January 2020 to March 2021. We used series of queries with changing keyword and period. Following is an example of one query:

*(“Google Trends”[Title/Abstract]) AND ((“2021/03/01”[Date - Publication]: “2021/03/31”[Date - Publication]))*

As a result of the first step, we have received a number of published papers containing the selected keyword in the title or abstract in the monthly resolution. In addition, we have also established a total number of published papers with identical conditions (keyword in title or abstract) before the year 2020. These numbers serve as starting point for further calculations.

In the second step, we have collected data with the following settings in Google Trends. We have set a time span since 2004 and used monthly data resolution for the years 2020 and 2021. We have selected the region worldwide and set all categories. The search was conducted using the index from Web Search. We have used three search topics: “*coronavirus”*, “*lockdown”*, “*social distancing”*. Searching for topics, instead of searching for particular search terms, helps cover the same topic in different countries and languages. Search topics cover all the keywords which are under the same topic. Setting the region worldwide allows seeing the same search topic's interest in all countries covered by Google search engine.

We have selected these search topics as the most discussed and presented in news and media. “*Coronavirus*” represents all keywords related to information about the disease [26]. “*Lockdown”* covers all keywords related to closing borders, economy, schools, and malls [5]. “*Social distancing*” refers to all keywords related to the use of personal protection measures, especially to keep the distance between two persons and using face masks [16].

## 3 Results

**Fig. 1:**
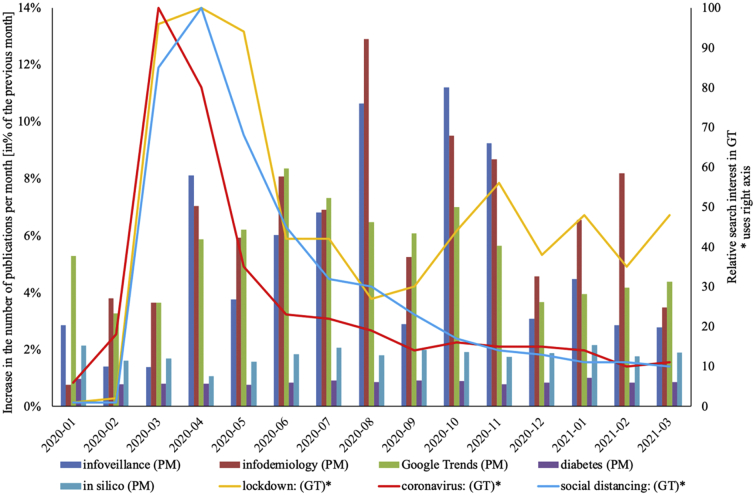
Increase in the number of publications per month in per cent of the total number in the previous month according to PubMed and a monthly search interest according to Google Trends (GT); PubMed and GT data accessed: 12/04/2021.

The relative increase in the number of publications compared to the previous month was examined using the PubMed database for the fifteen months starting January 2020. Our study with the PubMed database has shown a noticeable increase in the terms “*infodemiology*”, “*infoveillance*” and also high values for the term “*Google Trends*” in the title and abstract for scientific publications starting April 2020 compared to previous months (Fig. 1). The highest peak for “*infodemiology”* is noticed in August 2020, for “*infoveillance”* in October 2020, and for “*Google Trends”* in June 2020. During this investigation period, the relative increase in publications with the much broader term “*in silico*” and the increase in publications on the comparison term “*diabetes*” remained relatively constant.

Apart from the influence of the smaller number of publications with the terms “*infodemiology*” and “*infoveillance*” compared to the number of publications with “*diabetes*”, which are orders of magnitude larger, the observed increase is nevertheless apparent.

This trend in PubMed follows the peaks of search interest in Google Trends in March and April, which are based on the worldwide COVID-19-related requirement of the population for “*lockdown*” and “*social distancing*” (Fig. 1). First, the interest in searches increased; second, the scientific outcome was published. The search terms were queried in Google Trends between January 2020 and March 2021 in monthly resolution (Fig. 1). The highest interest of the search keyword is expressed as 100, while the lack of interest or insufficient data is expressed as 0. Volumes of searches are not related to each other. Each GT inquiry was made separately.

## 4 Discussion

Google Trends data are widely used for current research work on coronavirus disease. It delivers up-to-date relative search volumes for almost every country where COVID-19 was reported. For the past decade, data from Google Trends served as a source to conduct research on seasonality in infectious diseases, mental health, other diseases, and general population behaviour [19].

The main finding of this study is confirmation of the increase in the number of papers on infodemiology and infoveillance with the use of PubMed data during COVID-19 pandemic time. As presented results showed, in the period from January 2020 to March 2021, there is a relative increase in the number of published papers in the PubMed database. Our hypothesis that after increased interest in searches during the pandemic's initial months came an increased number of published papers on topics such as infoveillance, infodemiology and Google Trends is supported.

Data from Google Trends are used for different study fields. Here are only a few identified in previous studies regarding COVID-19 disease and its consequences. First, data from GT are used to predict COVID-19 incidences or to communicate risks [13]. An increasing number of studies covering occurrence and risks appearing in different countries was published. Second, the initial reports describing the coronavirus were mainly dominated by the presence of cough, difficult breathing, and fever. GT reveals, *e.g.*, interest in various other coronavirus symptoms, like loss of taste and smell, before these symptoms were identified as typical for infection [29]. Third, GT is used to measure interest in non-pharmaceutical interventions, *e.g.* personal protection measures like protection masks, hand washing, social distancing and mobility restrictions [15,27].

Fourth, it is used to assess current psychological effects and mental health on lockdowns and overall well-being. There is a reported increased number of searches like boredom, loneliness, worry and sadness [5]. Fifth, GT compares the present COVID-19 connection with other diseases like Kawasaki disease or bowel disease [6,20,25]. Google search data help to quickly discover the relation and magnitude between other know diseases and COVID-19. Sixth, it is used to assess the impact of COVID-19 on different treatment types like scheduled surgeries and telehealth applications and overall hospital care accessibility during the pandemic time [2]. Seventh, there is a significant impact of COVID-19 on the labour market visible in Google Trends searches [7]. Lockdown, mobility restrictions, closure of different economic sectors like tourism, hotels, and restaurants have a significant impact on the labour market.

A further literature review may identify several other fields. The most exciting field seems to be the **prediction** area. There are several studies where authors argue that Google Trends data can predict infections and the development of the disease [4]. On the other hand, some studies contribute the opposite [3]. The current pandemic state caused a new environment to conduct studies using methods known from “infodemiology” and “infoveillance”. Thus, many researchers applied Google Trends data in their research fields to conduct investigations in a global pandemic situation.

This study has some limitations. First, Google's popularity varies globally. For example, the search engine is not very popular in Russia, China, Japan, and South Korea. In the rest of the world, Google has more than half of the search market share. Therefore, for regions where Google has a higher market share, the results may be more representative. Second, by selecting the keyword “*diabetes”* for search through PubMed, we may have overlooked more suitable terms introduced through literature.

In a One Health approach, in addition to focusing on the health effects of the human population, it is also important to consider implications for and interactions with nature and the environment. In particular, the various corona viruses with their zoonotic origins teach us not to neglect veterinary public health issues [9,24]. As [11] describe, the health of humans, animals, and ecosystems should be reconnected, and Google Trends data can also provide a usable database for research on nature conservation issues [11,30]. From a One Health perspective, existing data can therefore be ideally combined and evaluated in a multidisciplinary approach in further studies and thus go beyond the disease focus. For example, Big Data are available from PubMed and Google Trends, which enable a variety of multifactorial evaluations and are to be used more intensively in the future. The present work exemplarily shows the use of several data sources and provides insights for further studies as well as for a better knowledge of how Google Trends takes health and medical search terms into account during a pandemic situation. Therefore, it creates a basis for future research on epidemic data with information from search engines and databases. In summary, it can be suggested that, in addition to the scientific challenges of the coronavirus crisis itself, the current situation could favour the publication of papers as a positive factor, which is based on contactless data analysis *in silico*.

## Declaration of Competing Interest

The authors declare no conflicts of interest.
